# Additional diagnostic value of tumor markers in cytological fluid for diagnosis of non-small-cell lung cancer

**DOI:** 10.1186/1471-2407-12-392

**Published:** 2012-09-07

**Authors:** Jin Hur, Hye-Jeong Lee, Ji Eun Nam, Young Jin Kim, Yoo Jin Hong, Hee Yeong Kim, Se Kyu Kim, Joon Chang, Joo-Hang Kim, Kyung Young Chung, Hye Sun Lee, Byoung Wook Choi

**Affiliations:** 1 Department of Radiology and Research Institute of Radiological Science, Severance Hospital, Yonsei University College of Medicine, Seoul, Republic of Korea; 2 Department of Internal Medicine, Severance Hospital, Yonsei University College of Medicine, Seoul, Republic of Korea; 3 Yonsei Cancer Center, Severance Hospital, Yonsei University College of Medicine, Seoul, Republic of Korea; 4 Department of Thoracic and Cardiovascular Surgery, Severance Hospital, Yonsei University College of Medicine, Seoul, Republic of Korea; 5 Department of Biostatistics, Severance Hospital, Yonsei University College of Medicine, Seoul, Republic of Korea; 6 Department of Radiology, Severance Hospital, Yonsei University College of Medicine, 250 Seongsanno, Seodaemun-gu, Seoul, 120-752, Republic of Korea

**Keywords:** Cytokeratin 19 fragments (CYFRA 21–1), Carcinoembryonic antigen (CEA), Squamous cell carcinoma antigen (SCC), Needle aspiration biopsy (NAB), Tumor marker, Cytological fluid, Non-small cell lung cancer (NSCLC)

## Abstract

**Background:**

Cytological fluid from a needle aspiration biopsy (NAB) is obtained directly from tumor tissue, therefore many biomarker candidates will be present in high concentrations. The aim of this study was to prospectively assess and validate the tumor markers CYFRA 21–1, CEA, and SCC in cytological fluid obtained from NAB samples to determine if they improved the performance of NAB for diagnosing non-small cell lung cancer (NSCLC).

**Methods:**

A total of 194 patients (M:F = 128:66, mean age 63.7 years) with suspected malignant pulmonary lesions were prospectively enrolled and underwent percutaneous NAB. Levels of CYFRA 21–1, CEA, and SCC were measured by immunoassay in serum and cytological fluid obtained during aspiration biopsy. Cut-off values to determined malignancy were 3.3 ng/mL in serum and 15.7 ng/mL in cytological fluid for CYFRA 21–1, 5 ng/mL and 0.6 ng/mL for CEA, and 2 ng/mL and 0.86 ng/mL for SCC.

**Results:**

Of 194 patients, 139 patients (71.6%) had NSCLC and 55 (28.4%) had benign lesions. Sensitivity increased significantly for NAB combined with cytological tumor markers compared with NAB alone (CYFRA 21–1: 95% versus 83.5%, p < 0.001, CEA: 92.1% versus 83.5%, p = 0.002, SCC: 91.4% versus 83.5%, p = 0.003). Accuracy improved significantly for NAB combined with cytological CYFRA 21–1 compared with NAB alone (95.9% versus 88.1%, p < 0.001). The area under curve (AUC) of NAB with cytological CYFRA 21–1 was significantly larger than for NAB alone (0.966 versus 0.917, p = 0.009).

**Conclusion:**

Of the tested tumor markers, cytological fluid measurements of CYFRA 21–1 improved the diagnostic performance of NAB for NSCLC.

## Background

Lung cancer has the highest mortality of any cancer worldwide and typically has a very poor prognosis. Lung cancer survival and therapy largely depend on the disease histology and stage at diagnosis [1,2]. Therefore, early diagnosis is paramount to improving prognosis. In clinical practice, diagnostic tools usually used for lung cancer include computed tomography (CT), bronchoscopy, and sputum analysis, which all have limitations for early diagnosis of lung cancer. Therefore, biopsy with histopathological examination is usually used to confirm the diagnosis [2].

Percutaneous needle aspiration biopsy (NAB) of the lung is a relatively safe and accurate method for diagnosing lung lesions with a reported accuracy, ranging from 64% to 97%. Major complications are rare [3,4]; however, transthoracic needle biopsy of lung lesions has a false negative rate of up to 29% for diagnosis of malignancies [4,5], and results from non-specific benign tissue or insufficient tissue are not reliable for excluding malignancy. Patients with these types of biopsy results should have tissue re-sampling with biopsy or surgical resection, or close clinical and imaging follow-up [5].

Serum tumor markers have been extensively studied in lung cancer, but none are specific for detecting lung cancer. Several tumor markers, including cytokeratin 19 fragments (CYFRA 21–1), carcinoembryonic antigen (CEA), and squamous cell carcinoma antigen (SCC), have been investigated for diagnostic and prognostic value in non-small cell lung cancer (NSCLC) [6-9]. Lung cancer tumor markers are useful tools for the follow-up of cancer patients, and are used mainly for monitoring the efficacy of therapy and early detection of recurrence [10-12]. One of the main drawbacks of serum tumor markers is that high concentrations are usually found only when the disease is at an advanced stage [13,14]. Therefore, clinically detecting a lung tumor at an early stage with serum marker assays is difficult [13,15].

Novel biomarkers are urgently needed for early diagnosis and predicting treatment response and prognosis. Many types of samples are possible for this purpose, but cytological fluid from a NAB is obtained directly from tumor tissue, therefore many biomarker candidates will be present in high concentrations [16]. We preformed an initial study on cytological tumor markers that suggested additional evaluation of cytological tumor markers CYFRA 21–1, CEA and SCC would be valuable in improving sensitivity in diagnosis of NSCLC in patients undergoing NAB. Our previous results showed a significant increase in sensitivity and accuracy for NAB combined with cytological CYFRA 21–1 compared with NAB alone (100% versus 85.7%, p = 0.001; 97.8% versus 89%, p = 0.0209, respectively) [17]. We conducted this external validation study to prospectively assess and validate the tumor markers CYFRA 21–1, CEA, and SCC in cytological fluid obtained from NAB samples to determine if they improved the performance of NAB for diagnosing NSCLC compared to serum tumor markers.

## Methods

### Patient selection

The external validation study protocol was approved by the Institutional Review Boards and written informed consent was obtained from all patients.

**Figure 1:**
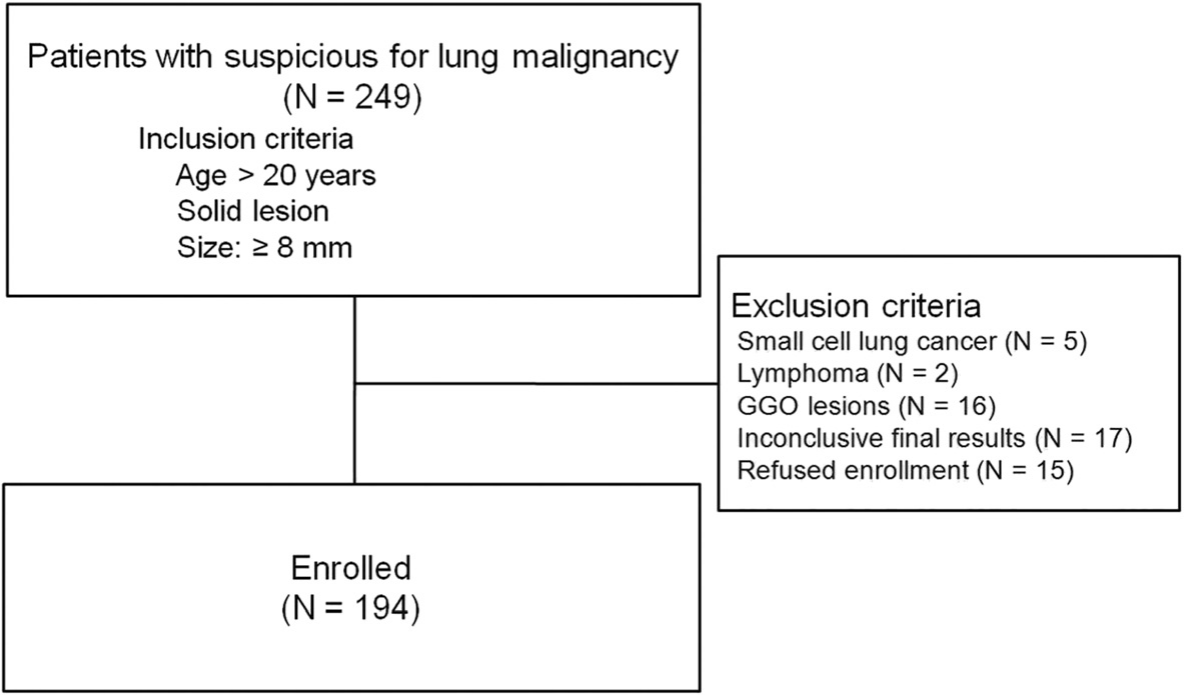
Flow chart of patient selection.

From November 1, 2009 to May 31, 2010, 249 patients who had a suspicious pulmonary nodule or mass concerning for lung malignancy on CT were prospectively enrolled for the validation arm. The inclusion criteria were age >20, lesion size more than 8 mm, and solid lesions (ground glass opacity (GGO) component of less than 50%). The exclusion criteria were: histologically-confirmed small cell lung cancer or lymphoma (n = 7), inconclusive pathological or cytological results (n = 17), GGO lesions (n = 16), and refusal to provide written informed consent (n = 15) (Figure 1).

Pre-biopsy evaluation included reviews of CT scans, laboratory studies, and medical records. All patients underwent either CT-guided NAB or fluoroscopy-guided NAB procedures, and all had NSCLC confirmed by histology and/or cytology. The final study population was comprised of 128 men and 66 women, aged from 34 to 82 years (mean age, 63.7 years). Data collection was systematized and a standardized registration form was prepared. For each patient, the following information was recorded: age, sex, history, biopsy site, size of the lesion, NAB results, pathology results, and laboratory data (serum and cytological fluid tumor markers for CYFRA 21–1, CEA, and SCC).

### Percutaneous transthoracic needle aspiration biopsy technique

Three experienced chest radiologists who had four, six, or ten years of experience performing thoracic biopsies performed the biopsy procedures. Fluoroscopy-guided biopsy interventions (n=52) were performed using a fluoroscope (Medix 130, Hitachi Med. Corp, Japan) and CT-guided biopsy interventions (n=142) were performed using a 16-channel multi-detector CT (MDCT) (Somatom Sensation 16, Siemens, Forchheim, Germany) equipped with CARE-vision software. The exposure parameters during CT-guided biopsy were 120 kV and 30 mA with a slice thickness of 6 mm. All procedures were performed with patients in a prone, supine, or lateral decubitus position, depending on the location of the lesion. The puncture area was cleaned with an antiseptic solution followed by administration of local anesthesia via subcutaneous injection of 1% lidocaine (Xylocaine, AstraZeneca). In all cases, more than two aspiration specimens were obtained to get enough specimen using 20–22 gauge Chiba needles (Cook, Bloomington, IN, USA). Cytological fluid was also aspirated during the procedure without additional needle punctures. Both aspiration specimens and cytological fluid were obtained with one needle puncture. A part of each specimen was placed in 95% ethyl alcohol for cytological examination. The rest of the specimen and fluid was prepared in tube for evaluation of cytological tumor marker.

Cytological results were evaluated and divided into the following diagnostic categories: ‘malignant’, ‘suspicious for malignancy’, ‘negative for malignancy’, and ‘non-diagnostic’ (e.g., cell paucity or samples with a few atypical cells). A designation of ‘malignancy’ or ‘suspicious for malignancy’ was considered a positive result. A designation of ‘negative for malignancy’ was considered a negative result. Non-diagnostic designations (n = 17) were considered neither positive nor negative, and the results were excluded from the analysis.

### Tumor marker analysis

Blood and cytological fluid were collected from each patient before any therapy. Serum and cytological fluid supernatants were obtained by centrifugation at 2000*g* for 10 min and stored at −40°C until tumor markers were assayed using commercial immunoassay kits. For both cytological fluid and serum, the technicians performing the assays were blinded to the final diagnosis. CYFRA 21–1 levels were measured using an electrochemiluminescent immunoassay (ECLIA) (CYFRA 21–1; Roche Diagnostics, Germany). CEA levels were measured using a chemiluminescence immunoassay (CLIA) (Centaur CEA; Bayer HealthCare, USA). SCC levels were measured using an immunoradiometric assay (IRMA) (SCC-RIABEAD; SRL Inc., Japan). Tumor markers in cytological fluid samples were assayed twice and mean values used for analysis.

For serum tumor marker levels, upper limits of normality were 3.3 ng/mL for CYFRA 21–1, 5 ng/mL for CEA and 2 ng/mL for SCC [18,19]. To the best of our knowledge, no other study measuring cytological tumor markers in NSCLC patients has been published. Therefore, no reference normal values for cytological fluid levels of various tumor markers were available. Therefore, we used cut-off values for cytological tumor markers of 15.7 ng/mL for CYFRA 21–1, 0.6 ng/mL for CEA, and 0.86 ng/mL for SCC [17]. In our previous study, receiver operating characteristics (ROC) curves were constructed using the values of tumor markers in the cytological fluid and a cut-off value was determined using the maximum Youden index for differentiation between malignant and benign lesions [17]. Any marker higher than the cut-off value was considered positive.

### Statistical analysis

A positive NAB result was considered a true-positive result if there was surgical confirmation, and a false-positive result if no evidence of malignancy was found during surgical resection (in the absence of preoperative chemotherapy). Results were considered negative if no tumor was identified during examination of the surgical specimen or regression was found on subsequent CT. Surgical confirmation of malignancy in the lesion with a negative NAB result was considered a false-negative finding.

Differences between the two groups (malignant and benign groups) were evaluated using the chi-square test or Fisher’s exact test. Sensitivity, specificity, accuracy, positive predictive value (PPV), and negative predictive value (NPV) of NAB alone and NAB combined with serum or cytological tumor markers (CYFRA21-1, CEA, and SCC) were calculated. When analyzing the diagnostic yield of a combination of tumor markers, a case was considered positive if either tumor marker or NAB result was positive, and negative if both tumor marker and NAB results were negative. Comparisons were made using weighted least squares (WLS) for statistical significance of sensitivity and accuracy between NAB combined with tumor markers and NAB alone [20]. To compare the performance of NAB alone and NAB combined with tumor markers, ROC curves were constructed and the area under the curve (AUC) was compared. Comparisons were made using the Delong method for statistical significance of AUC [21]. Statistical analyses were performed with SAS software (version 9.2 for Windows; SAS Institute, Cary, NC, USA) and p-values were adjusted using Bonferroni’s correction with less than 0.05 were considered statistically significant.

## Results

Among 194 patients, 139 patients (71.6%) had NSCLC and 55 (28.4%) patients had benign lesions. Table 1 summarizes patient characteristics. Histologic NSCLC types by patient and according to the WHO classifications were as follows [22]: 92 patients (66.2%) with adenocarcinoma (AC), 29 (20.8%) with squamous cell carcinoma (SQ), 3 (2.2%) with large-cell carcinoma, and 15 (10.8%) with NSCLC not otherwise specified (NOS). Of the 55 benign lesions, 29 were eventually diagnosed as benign based on progressive CT scans that showed lesion regression. Except for biopsy method and tuberculosis, all other characteristics, including age, sex, history of hypertension or diabetes, and smoking status were not significantly different between the two groups (p > 0.05).

**Table 1:** Demographic and baseline characteristics of 194 patients

**Characteristics**	**All (n=194)**	**Malignant (n=139)**	**Benign (n=55)**	**p-value**
**Sex**				0.811
Male	128(65.9)	91(65.5)	37(67.2)	
Female	66(34.1)	48(34.5)	18(32.8)	
**Age (years)**^a^	63.7±10.4	64.6±10.1	62.7±11.6	0.236
**Lesion Size (mm)**^a^	30.9±17.8	32.2±21.2	27.8±14.9	0.162
**Location**				0.385
Upper/Middle lobe	129(66.5)	95(68.3)	34(61.8)	
Lower lobe	65(33.5)	44(31.7)	21(38.2)	
**Method**				0.039
CT-guidence	142(73.1)	96 (69.1)	46(83.6)	
Fluoro-guidence	52(26.9)	43(30.9)	9(16.4)	
**Past history**				
Smoking	104(53.6)	74 (53.2)	30(54.5)	0.869
Hypertension	85(43.8)	59(42.4)	26(47.2)	0.283
Diabetes	25(12.8)	20(14.3)	5(9.1)	0.321
Pulmonary Tuberculosis	30(15.4)	16(11.5)	14(25.4)	0.015

Note-Values in parentheses are percentages. ^a^Data are presented as mean ± standard deviation.

The serum and cytological fluid levels of CYFRA 21–1, CEA, and SCC are presented in Table 2. CEA and SCC serum levels were significantly higher in the malignant group than in the benign group (p = 0.022, and p = 0.035, respectively). In the cytological fluid, CYFRA 21–1, CEA and SCC were significantly higher in the malignant group than in the benign group (p = 0.001, p = 0.003, and p = 0.027, respectively).

**Table 2:** Serum and Cytological Fluid Levels for CTFRA 21–1, CEA, and SCC in 194 Patients with Malignant and Benign Lesions

**Tumor marker**	**Malignant (n=139)**	**Benign (n=55)**	**p-value**
**Serum**			
**CYFRA 21–1 (ng/mL)**	8.36 ± 26.87	2.30 ± 1.23	0.097
**CEA (ng/mL)**	31.86 ± 93.37	2.67 ± 1.87	0.022
**SCC (ng/mL)**	1.22 ± 1.48	0.78 ± 0.62	0.035
**Cytological fluid**			
**CYFRA 21–1 (ng/mL)**	111.04 ± 159.59	5.01 ± 3.55	0.001
**CEA (ng/mL)**	20.63 ± 75.50	0.31 ± 0.48	0.003
**SCC (ng/mL)**	13.14 ± 33.76	2.84 ± 7.82	0.027

Note-Data are presented as mean ± standard deviation.

Table 3 describes the sensitivity, specificity, accuracy, PPV and NPV of NAB alone and NAB combined with serum or cytological tumor markers in 194 patients. Sensitivity and accuracy were not significantly different between NAB combined with serum tumor markers and NAB alone (for all three serum tumor markers, p > 0.05). However, sensitivity increased significantly for NAB combined with cytological tumor markers compared with NAB alone (CYFRA 21–1: 95% versus 83.5%, p < 0.001, CEA: 92.1% versus 83.5%, p = 0.002, and SCC: 91.4% versus 83.5%, p = 0.003). Accuracy improved significantly for NAB combined with cytological CYFRA 21–1 compared with NAB alone (95.9% versus 88.1%, p < 0.001). However, accuracy was not significantly different between NAB combined with cytological CEA or SCC and NAB alone (p = 0.138 and p > 0.999, respectively).

**Table 3:** Comparison of Diagnostic Results of NAB alone and NAB combined with Tumor Markers in 194 Patients

**Diagnostic Method**	**Sensitivity (%)**	**Specificity (%)**	**Accuracy(%)**	**PPV (%)**	**NPV (%)**
*NAB alone*	83.5	100.0	88.1	100.0	70.5
*NAB+Serum Tumor marker*					
CYFRA (3.3)	85.6	81.8	84.5	92.2	69.2
CEA (5)	86.3	90.9	87.6	96.0	72.5
SCC (2)	84.2	92.7	86.6	96.7	69.9
*NAB+Cytological Tumor marker*					
CYFRA (15.7)	95.0	98.2	95.9	99.2	88.5
CEA (0.6)	92.1	92.7	92.3	97.0	82.3
SCC (0.86)	91.4	70.9	85.6	88.8	76.5

Note-Numbers in parentheses are cut-off levels of tumor markers in ng/mL.

Abbreviations: PPV, positive predictive value; NPV, negative predictive value; CEA, carcinoembryonic antigen; SCC, squamous cell carcinoma; CYFRA, cytokeratin 19 fragments; NAB, needle aspiration biopsy.

**Figure 2:**
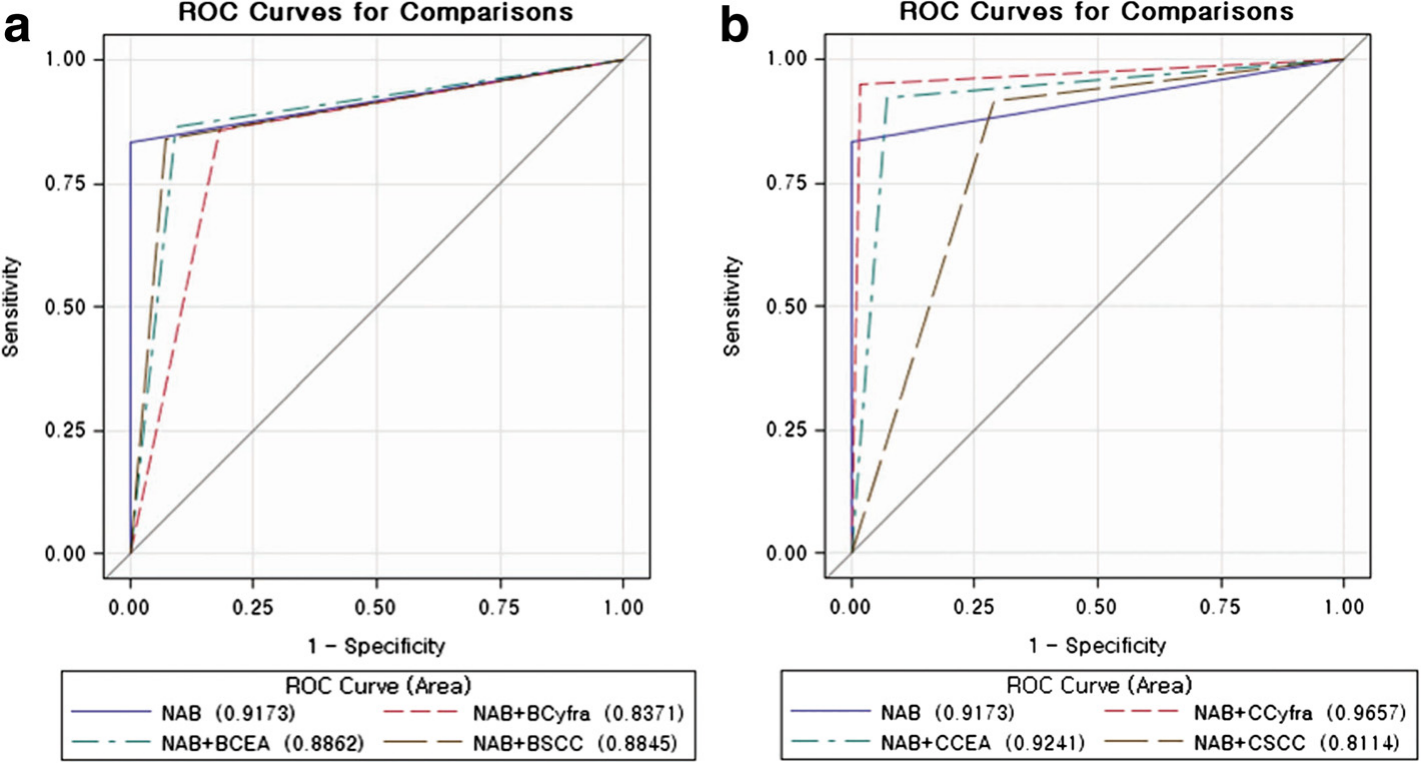
**Receiver operating characteristics (ROC) curves.** (**a**) ROC curves for NAB combined with CYFRA 21–1, CEA and SCC in the serum and NAB alone. (**b**) ROC curves for NAB combined with CYFRA 21–1, CEA and SCC in the cytological fluid and NAB alone.

For diagnosis of NSCLC, the AUC of NAB with serum CEA and SCC was not significantly larger than the AUC of NAB alone (p = 0.408, and p = 0.207, respectively). The AUC of NAB with serum CYFRA 21–1 decreased significantly compared with the AUC of NAB alone (p = 0.009). Using the cytological tumor markers, the AUC of NAB with cytological CYFRA 21–1 was significantly larger than the AUC of NAB alone (0.966 versus 0.917, p = 0.009). However, the AUC of NAB with cytological CEA was not significantly larger than the AUC of NAB alone (p = 0.999). The AUC of NAB with cytological SCC decreased significantly compared with the AUC of NAB alone (p = 0.003) (Table 4) (Figure 2).

**Table 4:** Comparison of Diagnostic Performance of NAB alone and NAB with Tumor Markers in 194 patients

**Tumor marker**	**AUC**	**P-value**
*NAB alone*	0.917 (95%CI;0.886-0.948)	
*NAB with Serum tumor marker*		
CYFRA (3.3)	0.837 (95%CI;0.778-0.896)	0.009
CEA (5)	0.886 (95%CI;0.838-0.934)	0.408
SCC (2)	0.885 (95%CI;0.838-0.901)	0.207
*NAB with Cytological tumor marker*		
CYFRA (15.7)	0.966 (95%CI;0.940-0.991)	0.009
CEA (0.6)	0.924 (95%CI;0.883-0.965)	0.999
SCC (0.86)	0.811 (95%CI;0.746-0.876)	0.003

Note-Numbers in parentheses are cut-off levels of tumor markers in ng/mL

Abbreviations: PPV, positive predictive value; NPV, negative predictive value; CEA, carcinoembryonic antigen; SCC, squamous cell carcinoma; CYFRA, cytokeratin 19 fragments; NAB, needle aspiration biopsy; AUC, areas under the curve.

Among the 139 malignant lesions, there were 42 cases of stage 1, 13 cases of stage 2, 16 cases of stage 3 and 68 cases of stage 4, respectively. In early stage (stage 1 and 2), the sensitivity and accuracy were not significantly different between NAB alone and NAB combined with serum tumor markers (for all three serum tumor markers, p > 0.05). However, sensitivity increased significantly for NAB combined with a cytological tumor marker compared with NAB alone (CYFRA 21–1: 94.4% versus 75.9%, p = 0.006, CEA: 87% versus 75.9%, p = 0.042, and SCC: 87% versus 75.9%, p = 0.042, respectively). Accuracy improved significantly for NAB combined with cytological CYFRA 21–1 compared with NAB alone (96.3% versus 88.1%, p = 0.021). In advanced stage (stage 3 and 4), the sensitivity and accuracy were not significantly different between NAB alone and NAB combined with serum tumor markers (for all three serum tumor markers, p > 0.05). However, sensitivity increased significantly for NAB combined with a cytological tumor marker compared with NAB alone (CYFRA 21–1: 95.2% versus 88.1%, p = 0.042, CEA: 95.2% versus 88.1%, p = 0.042, respectively). The accuracy was not significantly different between NAB alone and NAB combined with cytological tumor markers (for all three tumor markers, p > 0.05) (Table 5).

**Table 5:** Comparison of Diagnostic Results of NAB alone and NAB combined with Tumor Markers according to tumor stage

**Diagnostic Method**	**Sensitivity (%)**	**Specificity (%)**	**Accuracy (%)**	**PPV (%)**	**NPV (%)**
*Early Stage (Stage 1 and 2, n=55)*					
*NAB alone*	75.9	100	88.1	100	80.9
*NAB+Serum Tumor marker*					
CYFRA (3.3)	77.8	81.8	79.8	80.8	78.9
CEA (5)	75.9	90.9	83.5	89.1	79.4
SCC (2)	75.9	92.7	84.4	91.1	79.7
*NAB+Cytological Tumor marker*					
CYFRA (15.7)	94.4	98.2	96.3	98.1	94.7
CEA (0.6)	87	92.7	89.9	92.2	87.9
SCC (0.86)	87	70.9	78.9	74.6	84.8
*Advanced Stage (Stage 3 and 4, n=84)*					
*NAB alone*	88.1	100	92.8	100	84.6
*NAB+Serum Tumor marker*					
CYFRA (3.3)	90.5	81.8	87.1	88.4	84.9
CEA (5)	92.9	90.9	92.1	94	89.3
SCC (2)	89.3	92.7	90.6	94.9	85
*NAB+Cytological Tumor marker*					
CYFRA (15.7)	95.2	98.2	96.4	98.8	93.1
CEA (0.6)	95.2	92.7	94.2	95.2	92.7
SCC (0.86)	94	70.9	84.9	83.2	88.6

Note-Numbers in parentheses are cut-off levels of tumor markers in ng/mL.

Abbreviations: PPV, positive predictive value; NPV, negative predictive value; CEA, carcinoembryonic antigen; SCC, squamous cell carcinoma; CYFRA, cytokeratin 19 fragments; NAB, needle aspiration biopsy.

In the results of subgroup analysis for diagnosing adenocarcinoma (n=92), the sensitivity and accuracy were not significantly different between NAB alone and NAB combined with serum tumor markers (for all three serum tumor markers, p > 0.05). However, sensitivity increased significantly for NAB combined with a cytological tumor marker compared with NAB alone (CYFRA 21–1: 92.4% versus 79.3%, p = 0.003, CEA: 89.1% versus 79.3%, p = 0.009, respectively). Accuracy improved significantly for NAB combined with cytological CYFRA 21–1 compared with NAB alone (94.6% versus 87.1%, p = 0.006). In the results of subgroup analysis for diagnosing squamous cell carcinoma (n=29), the sensitivity and accuracy of NAB combined with any serum or cytological tumor marker were not significantly higher than NAB alone (for all serum and cytological tumor markers, p > 0.05) (Table 6).

**Table 6:** Comparison of Diagnostic Results of NAB alone and NAB combined with Tumor Markers according to histological subtype

**Diagnostic Method**	**Sensitivity(%)**	**Specificity(%)**	**Accuracy(%)**	**PPV (%)**	**NPV (%)**
*Adencarcinoma subtype (n=92)*					
*NAB alone*	79.3	100	87.1	100	74.3
*NAB+Serum Tumor marker*					
CYFRA (3.3)	81.5	81.8	81.6	88.2	72.6
CEA (5)	81.5	90.9	85	93.8	74.6
SCC (2)	79.3	92.7	84.4	94.8	72.9
*NAB+Cytological Tumor marker*					
CYFRA (15.7)	92.4	98.2	94.6	98.8	88.5
CEA (0.6)	89.1	92.7	90.5	95.3	83.6
SCC (0.86)	83.5	70.9	81.6	88	78
*Squamous cell carcinoma subtype (n=29)*					
*NAB alone*	89.7	100	96.4	100	94.8
*NAB+Serum Tumor marker*					
CYFRA (3.3)	93.1	81.8	85.7	73	95.7
CEA (5)	93.1	90.9	91.7	84.4	96.2
SCC (2)	93.1	92.7	92.9	87.1	96.2
*NAB+Cytological Tumor marker*					
CYFRA (15.7)	100	98.2	98.8	96.7	100
CEA (0.6)	96.6	92.7	94	87.5	98.1
SCC (0.86)	100	70.9	81	64.4	100

Note-Numbers in parentheses are cut-off levels of tumor markers in ng/mL.

Abbreviations: PPV, positive predictive value; NPV, negative predictive value; CEA, carcinoembryonic antigen; SCC, squamous cell carcinoma; CYFRA, cytokeratin 19 fragments; NAB, needle aspiration biopsy.

## Discussion

This study was designed to determine whether analysis of the tumor markers CYFRA 21–1, CEA, and SCC in cytological fluid could improve the performance of NAB in the diagnosis of NSCLC. Our external validation study showed that NAB with additional evaluation of cytological tumor marker of CYFRA 21–1 can improve sensitivity and accuracy in the diagnosis for NSCLC.

Early diagnosis of lung cancer is essential for increased survival, but this is difficult because the symptoms are non-specific and are frequently found in the risk group of smoker patients. In clinical practice, lung cancer diagnosis still depends primarily on imaging techniques, such as x-ray and CT, and if a suspicious lesion is present, biopsy with histopathological examination is used to confirm the diagnosis.

Currently, transthoracic NAB is often performed to obtain a definitive diagnosis and is a useful procedure for diagnosing pulmonary nodules that are highly likely to be malignant, especially in patients who are not candidates for surgery [5]. Although NAB of the lung is a relatively safe and accurate method for diagnosing lung lesions, the results of transthoracic needle biopsy of lung lesions often results in false negative in the diagnosis of malignancy, and non-specific results are common in NAB [5]. Previous reports found that, transthoracic needle biopsy of lung lesions has a false negative rate of up to 29% for malignancy diagnosis [4,5], and 27% of the non-specific results are reported to later turn out to be malignant [23]. Therefore, patients with suspected lung malignancy but inconclusive results on initial lung biopsy often require a second transthoracic lung biopsy or surgical biopsy including video-assisted thoracoscopic surgery (VATS).

Currently, several tumor markers in the serum have been extensively studied in lung cancer, but none is specific for diagnosis of NSCLC. Tumor markers may aid in clinical diagnosis as well as prognosis and follow-up. Tumor marker determination is a simple, inexpensive test, available in most centers where lung cancer is diagnosed. However, the results to date do not support the widespread use of tumor markers for diagnosis.

We used cytological fluid obtained by biopsy as a new type of sample for tumor marker analysis. We hypothesized that cytological fluid obtained from NAB has the potential to be an effective sample, as it is obtained directly from tumor tissue, and because many biomarkers candidates will exist in high concentrations [16]. Although lung cancer tissue biopsy is invasive, biopsy and resection are currently the gold standards for confirmative diagnosis, and thus are generally performed in cases of high suspicion of malignancy. Furthermore, performing the extra step of measuring concentrations of tumor markers in the fluid that is aspirated does not require an additional puncture, takes little extra time, and is easy.

Results from our previous study indicated that additional evaluation of tumor markers in the cytological fluid can improve the diagnostic performance of CT-guided NAB in NSCLC patients. Our previous results showed a significant increase in sensitivity and accuracy for NAB combined with CYFRA 21–1 compared with NAB alone (100% versus 85.7%, p = 0.001; 97.8% versus 89%, p = 0.0209, respectively) [17]. This was supported by the results of this validation study. When we combined the results of serum tumor markers, sensitivity and accuracy were not significantly different between NAB combined with serum tumor markers and NAB alone (for all three serum tumor markers, p > 0.05). However, sensitivity increased significantly for NAB combined with any cytological tumor marker compared to NAB alone (95% for CYFRA 21–1, p < 0.001; 92.1% for CEA p = 0.002; and 91.4% for SCC, p = 0.003). The accuracy improved significantly for NAB combined with cytological CYFRA 21–1 compared with NAB alone (95.9% versus 88.1%, p < 0.001). When we compared the AUC between NAB combined with tumor markers and NAB alone, the AUC of NAB with cytological CYFRA was significantly larger than the AUC of NAB alone (0.966 versus 0.917, p = 0.009). Furthermore, in subgroup analysis, the sensitivity also increased significantly for NAB combined with any cytological tumor markers compared with NAB alone in early stage lung cancer. This indicated that this cytological tumor marker had additional value in the diagnosis of NSCLC. According to our validation study, NAB combined with cytological CYFRA 21–1 had the best diagnostic performance, similar to findings of previous studies [6,10]. CYFRA is the most sensitive tumor marker for NSCLC. Wieskopf et al. [20] reported that serum CYFRA 21–1 was a sensitive and specific tumor marker for NSCLC diagnosis, appearing more sensitive and more specific than other tumor markers such as CEA and SCC.

In the results of subgroup analysis according to histological cell types, the sensitivity and accuracy of NAB combined with cytological CYFRA 21–1 was significantly higher than NAB alone for diagnosing adenocarcinoma. Although CYFRA is one of the most sensitive tumor markers available, its relationship with specific histology is controversial. Some studies reported that CYFRA has no clear relationship to different histological cell subtypes in NSCLC, whereas, others have reported that CYFRA is a more sensitive and specific tumor marker especially for the squamous cell subtype [6,10,20]. Previous studies using immunohistochemical analysis for resected adenocarcinoma and squamous cell carcinoma, showed that cytokeratin-19, the marker used for CYFRA21-1, stained both adenocarcinoma and squamous cell carcinoma strongly and indiscriminately [24][25]. Although NAB combined with cytological CYFRA 21–1 had tendency to increase sensitivity and accuracy for diagnosing squamous cell carcinoma subtype, the sensitivity and accuracy were not significantly different between NAB alone and NAB combined with cytological CYFRA 21–1. This may be explained in that the sample size for squamous cell carcinoma subtype was small. In our study, more than half of the cases were adenocarcinomas, and only 20% were squamous cell carcinomas.

Based on our validation study, cytological fluid could be an effective sample for tumor markers and may be clinically useful in lung cancer diagnosis. This is because while lung biopsy using needle aspiration is a confirmative method for lung cancer diagnosis, 29% of NAB results can be non-diagnostic [18,19]. Taking the extra step of measuring tumor markers concentrations in the aspirated fluid does not require an additional puncture, takes little extra time, and is easy to perform. Therefore, we believe that in cases of suspicious malignant nodules or masses showing a negative or inconclusive cytological result, determining tumor markers in the cytological fluid may be a helpful complementary tool for lung cancer diagnosis.

Our study has some limitations. First, the major two histologic types were not represented equally. More than half of the cases were adenocarcinomas, and only 20% were squamous cell carcinomas. Furthermore, in subgroup analysis, the sensitivity increased significantly for NAB combined with cytological tumor markers (CYFRA 21–1 and CEA) compared with NAB alone in the diagnosis of adenocarcinoma, whereas, the sensitivity and accuracy were not significantly different between NAB alone and NAB combined with any serum or cytological tumor markers for diagnosing squamous cell carcinoma. Therefore, the value of cytological tumor markers determined in this study might be limited for cell types other than adenocarcinoma. Second, the results may be influenced by the method used to choose the cut-off point. Although serum tumor markers have normal reference values, no reference normal values are available for cytological fluid levels of the tumor markers. In this study, we used the cut-off values for cytological tumor markers determined in our previous study to validate the cut-off values selected for tumor markers as an independent series. In our previous study, ROC curves were constructed using tumor marker values in the cytological fluid and a cut-off value was determined using the maximum Youden index for differentiation between malignant and benign lesions. Third, while most lesions had histopathologically confirmed diagnoses, 29 lesions required follow-up imaging studies and clinical examinations; the follow-up time used to classify lesions as benign was at least 12 months using imaging finding for lesion regression.

## Conclusion

In conclusion, among the tumor markers studied, CYFRA 21–1 measurements in cytological fluid improved the diagnostic performance of percutaneous NAB for NSCLC. Our results provide a rationale for evaluating tumor markers in cytological fluid as complementary to NAB for lung cancer diagnosis. Therefore, in cases of suspicious malignant nodules or masses showing a negative or inconclusive cytological result, determining tumor markers in cytological fluid may be helpful for lung cancer diagnosis.

## Abbreviations

AUC: Area under the curve; CEA: Carcinoembryonic antigen; CYFRA 21–1: ; NAB: Needle aspiration biopsy; NSCLC: Non-small cell lung carcinoma; ROC: Receiver operating characteristic; SCC: Squamous cell cancer; VATS: Video-assisted thoracoscopic surgery.

## Competing interest

Jin Hur: No disclosures, Hye-Jeong Lee: No disclosures, Ji Eun Nam: No disclosures, Young Jin Kim: No disclosures, Yoo Jin Hong: No disclosures, Hee Yeong Kim: No disclosures. Se Kyu Kim: No disclosures, Joon Chang: No disclosures, Joo-Hang Kim: No disclosures, Kyung Young Chung: No disclosures, Hye Sun Lee: No disclosures, Byoung Wook Choi: No disclosures.

## Authors’ contributions

JH Study design, Literature search, Data collection, Data analysis, Data interpretation, Writing, Funding. HJL: Literature search, Data collection, Data analysis. JEN: Literature search, Data collection, Data analysis, YJK: Literature search, Data collection, Yoo Jin Hong: Literature search, Data collection. HYK: Literature search. Data collection. SKK: Data collection, JC: Data collection. JHK: Data collection. KYC: Data collection. Hye Sun Lee: Data analysis, Statistical analysis. BWC: Study design, Literature search, Data collection, Data analysis, Data interpretation, Writing, editing, Funding. All authors read and approved the final manuscript.

## Pre-publication history

The pre-publication history for this paper can be accessed here:

http://www.biomedcentral.com/1471-2407/12/392/prepub
